# Mothers’ perception of recovery and satisfaction with patent medicine dealers’ treatment of childhood febrile conditions in rural communities

**DOI:** 10.1186/s12936-016-1384-5

**Published:** 2016-06-28

**Authors:** Georgian Chiaka Ibeneme, Ada Caroline Nwaneri, Sam Chidi Ibeneme, Pauline Ezenduka, Vanessa Strüver, Gehard Fortwengel, Ifeoma Joy Okoye

**Affiliations:** Department of Nursing Sciences, Faculty of Health Sciences and Technology, College of Medicine, Ebonyi State University, Abakaliki, Ebonyi State Nigeria; Department of Nursing Sciences, Faculty of Health Sciences and Technology, College of Medicine, University of Nigeria, Enugu Campus, Abakaliki, Ebonyi State Nigeria; Department of Medical Rehabilitation, Faculty of Health Sciences and Technology, College of Medicine, University of Nigeria, Enugu Campus, Abakaliki, Ebonyi State Nigeria; Department of Nursing Sciences, Faculty of Health Sciences and Technology, College of Medicine, Nnamdi Azikiwe University, Nnewi, Anamabra State Nigeria; German UNESCO Unit on Bioethics, Fakultät III-Medien, Information und Design, Hochschule Hannover-University of Applied Sciences and Arts, Hannover, Germany; Department of Radiation Medicine, Faculty of Medical sciences, College of Medicine, University of Nigeria, Enugu Campus, Abakaliki, Ebonyi State Nigeria

**Keywords:** Patent medicine dealers, Childhood febrile conditions, Mothers’ perception of recovery, Mothers’ satisfaction, Rural community

## Abstract

**Background:**

Infant mortality in rural areas of Nigeria can be minimized if childhood febrile conditions are treated by trained health personnel, deployed to primary healthcare centres (PHCs) rather than the observed preference of mothers for patent medicine dealers (PMDs). However, health service utilization/patronage is driven by consumer satisfaction and perception of services/product value. The objective of this study was to determine ‘mothers’ perception of recovery’ and ‘mothers’ satisfaction’ after PMD treatment of childhood febrile conditions, as likely drivers of mothers’ health-seeking behaviour, which must be targeted to reverse the trend.

**Methods:**

Ugwuogo-Nike, in Enugu, Nigeria, has many PMDs/PHCs, and was selected based on high prevalence of childhood febrile conditions. In total, 385 consenting mothers (aged 15–45 years) were consecutively recruited at PMD shops, after purchasing drugs for childhood febrile conditions, in a cross-sectional observational study using a pre-tested instrument; 33 of them (aged 21–47 years) participated in focus group discussions (FGDs). Qualitative data were thematically analysed while a quantitative study was analysed with Z score and Chi square statistics, at p < 0.05.

**Results:**

Most participants in FGDs perceived that their child had delayed recovery, but were satisfied with PMDs’ treatment of childhood febrile conditions, for reasons that included politeness, caring attitude, drug availability, easy accessibility, flexibility in pricing, shorter waiting time, their God-fearing nature, and disposition as good listeners. Mothers’ satisfaction with PMDs’ treatment is significantly (p < 0.05) associated with mothers’ perception of recovery of their child (χ^2^ = 192.94, df = 4; p < 0.0001; Cramer’s V = 0.7079). However, predicting mothers’ satisfaction with PMDs’ treatment from a knowledge of mothers’ perception of recovery shows a high accord (lambda_[A from B]_ = 0.8727), unlike when predicting mothers’ perception of recovery based on knowledge of mothers’ satisfaction with PMDs’ treatment (lambda_[A from B]_ = 0.4727).

**Conclusions:**

Mothers’ satisfaction could be the key ‘driver’ of mothers’ health-seeking behaviour and is less likely to be influenced by mothers’ perception of recovery of their child. Therefore, mothers’ negative perception of their child’s recovery may not induce proportionate decline in mothers’ health-seeking behaviour (patronage of PMDs), which might be influenced mainly by mothers’ satisfaction with the positive attributes of PMDs’ personality/practice and sets an important agenda for PHC reforms.

## Background

Preference for the services of patent medicine dealers (PMDs) in the treatment of childhood febrile conditions is common in rural [1] and urban areas [2]. The choice to patronize PMDs in the treatment of childhood febrile conditions (fever) has been shown to depend on a number of incentives, including geographical accessibility, shorter waiting times, more reliable drug stocks, longer opening hours, greater confidentiality, more personable social interactions, ease of seeking advice, low cost of services and flexible pricing system, and no separate fee charged for consultation [3]. Invariably, the business model operated by PMDs seems to target client satisfaction to ensure loyalty and patronage by incentivizing services offered. This might explain why PMDs are preferentially patronized in childhood febrile conditions rather than primary healthcare centres (PHCs) by mothers in rural areas. It was considered that first responders to childhood febrile conditions are mothers who take their children for treatment, and that therefore ‘mothers’ perception of recovery’ and ‘mothers’ satisfaction’ with PMDs’ treatment of their child’s febrile conditions could be important drivers to explain mothers’ health-seeking behaviour in patronizing PMDs in rural areas. It is important, in setting a health policy agenda, to reverse this trend, which holds adverse implications for infant mortality rates, because PMDs are not formally trained to diagnose and treat any form of diseases [4].

PMDs can be described as persons without formal pharmacy training who sell orthodox pharmaceutical products on a retail basis for profit [4]. Although they have been shown to have little formal health training, they are usually the first choice in healthcare and a recognized primary source of orthodox drugs in Nigeria for both rural and urban populations, especially the poor [1]. The choice to patronize PMDs in the treatment of childhood febrile conditions (fever) has been shown to depend on a number of incentives already mentioned [3]. This might explain the observed preference for PMDs’ services by rural dwellers [1], even where there are quality and affordable healthcare services at PHCs. It is possible that by using incentives as a business strategy, PMDs have positively influenced mothers’ satisfaction, which would have implications for mothers’ health-seeking behaviour in childhood febrile conditions. It was considered that mothers’ negative perception of recovery by their child might translate to mothers’ dissatisfaction after PMDs’ treatment, since PMDs are untrained in managing childhood febrile conditions. Invariably, if this were the case, the observation that mothers in rural communities prefer to patronize the PMDs in childhood febrile conditions would not arise. Therefore, the relationship between mothers’ perception of recovery, and mothers’ satisfaction after treatment of childhood febrile conditions by PMDs, needs to be explored to gain a fuller understanding of how these factors interact as important drivers of mothers’ health-seeking behaviour in rural communities. An understanding of mothers’ health-seeking behaviour can be deployed to improve patronage of rural PHCs in childhood febrile conditions, and eventually reverse the trend in infant mortalities that may arise from treatment practices of PMDs in rural communities.

Human perception can be affected by factors, such as learning, experience and emotion, among others [5]. Invariably, ‘satisfactory feeling’ or emotion can affect perception and consequently influence human behaviour. Mothers’ perception of recovery can be influenced by mothers’ satisfaction with treatment of childhood febrile conditions in a manner that may evoke positive consumer attitude [6] and patronage of PMD services. Previous authors [7, 8] identified some incentives that are likely to influence patient satisfaction, including caring attitude, technical quality care, accessibility and convenience, finance, physical environment, availability, efficacy and outcome of treatment. This implies that incentivizing health services is an important issue for consumers to influence their health-seeking behaviour, such that in spite of the availability of healthcare facilities/and qualified personnel in rural PHCs, mothers still preferred to patronize PMDs [9] regardless of PMDs’ little or no formal health training [1, 10]. It is plausible that incentives in treatment services and treatment outcome (measured using mothers perception of recovery), are two key factors that may influence mothers’ satisfaction and drive mothers’ health-seeking behaviour. It was hypothesized that mothers’ satisfaction with PMD treatment of childhood febrile conditions is in strong accord with mothers’ perception of recovery of their child after treatment by PMDs. This is of interest to health policy makers, administrators and practitioners|, and was investigated. The major objective of the study is to determine mothers’ perception of recovery and mothers’ satisfaction with PMDs’ treatment of childhood febrile conditions in a rural community, and their relationships as determinants of mothers’ health-seeking behaviour. Ugwuogo-Nike community was chosen for this study, because several cases of childhood febrile conditions have been reported in that locality, which warranted studies on PHCs and PMDs in that community [11, 12], and therefore easily situates the findings of this study in contemporary literature for easy comparison.

## Methods

### Population and study design

A descriptive, cross-sectional survey design was used to study 385 mothers in the rural community of Ugwuogo-Nike, Enugu State, Nigeria to examine the inter-relationships between mothers’ perception of recovery and mothers’ satisfaction with PMD treatment of childhood febrile conditions. Recruitment was conducted at five central PMD shops in the community. The process involved three stages: obtaining informed consent, administering the questionnaire and conducting verbal interviews. The questionnaire included information on socio-demographics, occupation, history of child’s febrile illness, and other medical history of immunization. Participants gave their written informed consent prior to data collection, and ethical approval from the University of Nigeria Health Research Ethics Committee was obtained NHREC/05/01/2008B. All data collated were de-identified for analysis.

### Data collection

Data for this study were collected using an interviewer-administered questionnaire designed specifically for this study. Questions were generated from the literature relating to mothers’ perception of recovery from childhood febrile conditions after treatment by PMDs. The questions were designed in a simple English language with the intention of eliciting answers for the formulated research questions. The interview guide consisted of two sections: Section A consisted of demographic information of subjects, while Section B was made up of information on mothers’ perception of recovery and mothers’ satisfaction after treatment of childhood febrile condition by PMDs. The instrument was pre-tested in a pilot study on 20 mothers (chosen from Akpuoga-Nike community which was not included in the study but has similar geographical characteristics as the area of study) and was found to have a reliability of 0.87. Face and content validity of the instrument was determined by three experts. Mothers’ perception of recovery was categorized, based on the five key identified views expressed by mothers from the pilot study, as: (a) child fully recovered; (b) fever persisted; (c) fever relapsed; (d) recovered with disability; and, (e) child died.

With the test instrument, mothers’ perception of recovery was obtained from respondents and then entered and verified by the researchers after further questioning during the verbal interview. Four inclusion criteria were applied as follows: (1) mothers who had one or more children under the age of 5 years who had previously had fever; (2) mothers who used the services of PMDs in the treatment of childhood fever; (3) mothers living with their child and involved in taking care of their child during fever episodes; and, (4) mothers who were emotionally and mentally stable at the time of the study.

The target population of the study was mothers whose children were under 5 years old and had been treated by PMDs for childhood febrile conditions. As this population was unknown, sample size determination was based on reports by Goodman et al. [10] that show that the number of mothers that used PMDs in childhood malaria ranged from 15 to 82 %, with a median around 50 %. The prevalence rate for health-seeking behaviour was thus taken as 50 % in order to have a representative study sample, which was determined as (n) = 384, according to Colditz et al. [13]. For equal representation, 385 respondents were recruited from the five recruitment centres (i.e. each centre recruited 77 respondents) used for the study. Thus, consenting respondents were consecutively recruited at the major PMD shops serving the five villages: Umunonu, Umunameze, Umunagbo, Amakpaka, and Obinagu, of the Ugwuogo-Nike community. These respondents were mothers who came to patronize PMDs for treatment of childhood febrile conditions. The researchers had the permission of the PMDs to observe the interactions with their clients, who came to buy desired drugs. On each occasion, the researchers approached and recruited consenting mothers who purchased drugs for childhood febrile conditions. Each respondent was listed under their corresponding villages until the desired number for each village was achieved.

To appraise the point of mothers’ utilization of PMDs in the treatment algorithm of childhood febrile conditions, the following question was asked: “At what point in time did you, as a mother in the rural area of Ugwuogo-Nike, resort to PMDs in the treatment of childhood febrile condition? To measure mothers’ perception of recovery after treatment by PMDs, respondents were asked: “What is your perception of recovery of your child from childhood febrile conditions after treatment by PMDs in the rural areas of Ugwuogo-Nike?” To measure mothers’ satisfaction after treatment by PMDs, participants were asked: “What is your view on the satisfaction of treatment given by PMDs in childhood febrile condition?”

With this information, it was possible to determine the relationship between mothers’ perceptions of recovery and mothers’ satisfaction after treatment of childhood febrile conditions by PMDs in the rural areas of Ugwuogo-Nike. To provide an in-depth grasp of the issues already explored using the pre-tested questionnaire, further information on mothers’ perception and mothers satisfaction with PMDs’ treatment of childhood febrile conditions was obtained in focus group discussion (FGD) using a structured interview guide. The FGDs involved women who met the selection criteria, and who were purposively selected from the respondents. Five FGDs (one for each of the five villages of Ugwuogo-Nike) were held with five to seven mothers, each lasting for 50–55 min. In the FGDs, mothers’ perceptions and mothers’ satisfaction with PMDs’ treatment were explored in order to gain insight of the variable factors that may influence mothers’ health-seeking behaviour in childhood febrile conditions. Verbatim responses from the FGDs were transcribed and categorized into different themes, including mothers’ perceptions and mothers’ satisfaction as well as variables which in their opinion might influence their perception and level of satisfaction in this context.

### Statistical analysis

All data analyses were performed using faculty Vassar computational software (USA). Mothers’ perception of recovery was categorized, based on the five key identified views expressed by mothers from the pilot study, as: child fully recovered; fever persisted; fever relapsed; recovered with disability; and, child died. Mothers’ satisfaction was labelled as: satisfied and not satisfied. Item-by-item analysis was carried out to show the response frequency and percentages of various categories of data. Data obtained from structured interview guide were collated, tallied and analysed using Z score to determine the significance of the difference between two independent proportions. Chi square was also applied to determine the association between the variables; Crammer’s V was used to determine the strength of the relationship, while lamda was used to predict the accord between mothers’ perception of recovery and mothers’ satisfaction. All statistical tests were two-sided, and significance was set at p < 0.05.

## Results

Of the 385 respondents, 133 (34.5 %) were aged between 15 and 24 years, 137 (35.6 %) were between 25 and 34 years old; 92 (23.9 %) were 35–44 years old, and 23 (6.0 %) were 45 years and above (Table 1); 286 (74.3 %) respondents were married, 48 (12.5 %) were single, 44 (11.4 %) were widows, and seven (1.8 %) were divorcees; 367 (95.3 %) respondents were Christian, three (1.8 %) were Moslem, 15 (3.9 %) belonged to other religions; 47 (12.2 %) respondents had no formal education, 142 (36.9 %) had primary school education, 162 (42.1 %) had secondary school education, 34 (8.8 %) had tertiary school education.

**Table 1:** Demographic data of the respondents

	Frequency	Percentage (%)
Age (years)		
15–24	133	34.5
25–34	137	35.6
35–44	92	23.9
45 and above	23	6.0
Total	385	100.0
Marital status		
Married	286	74.3
Single	48	12.5
Widow	44	11.4
Divorcee	7	1.8
Total	385	100.0
Religion		
Christianity	367	95.3
Islam	3	0.8
Others specify	15	3.9
Total	385	100.0
Highest level of education		
None	47	12.2
Primary education	142	36.9
Secondary education	162	42.1
Tertiary education	34	8.8
Total	385	100.0
Parity		
1.00	56	14.6
2.00	87	22.6
3.00	87	22.6
4.00	61	15.8
5.00	35	9.1
6.00	34	8.8
7.00 and above	25	6.49
Total	385	100
Occupation		
House wife	24	6.2
Farming	142	37.0
Civil servant	40	10.3
Trading	112	29.0
Tailoring	54	14.0
Others specify	13	3.3
Total	385	100

The proportion of mothers who perceived their child had recovered fully (143, 37.14 %) was significantly smaller (Z = −7.1354, p ≤ 0.0001) than those who perceived their child did not recover fully (242, 62.86 %), which included mothers who perceived that their child had persistent fever (25, 6.49 %), relapsed fever (102, 26.49 %), recovered with disability (53, 13.77 %), died (62, 16.10 %) (Table 2). However, a significantly higher proportion (Z = 11.604, p ≤ 0.0002) of mothers (273, 70.99 % relative to 112, 29.10 %) were satisfied with the treatment given to their child by PMDs (Table 3). A significantly higher proportion (Z = 8.2680, p ≤ 0.0001), or 137/143 mothers who perceived that their child recovered fully, were satisfied with the treatment from PMDs in childhood febrile conditions, relative to mothers who were not satisfied (Table 4). In contrast, a significantly higher proportion (Z = 8.2320, p ≤ 0.0002) or 45/62 mothers who reported that their child died were not satisfied with PMDs’ treatment of childhood febrile conditions, relative to 17/62 mothers who were satisfied. Mothers’ satisfaction with treatment is significantly (p < 0.05) associated with mothers’ perception of recovery of their child (χ^2^ = 192.94, df = 4; p < 0.0001; Cramer’s V = 0.7079). However, predicting mothers’ satisfaction with treatment from a knowledge of mothers’ perception of recovery, shows a high accord (lambda_[A from B]_ = 0.8727), unlike when predicting mothers’ perception of recovery based on knowledge of mothers’ satisfaction with the treatment (lambda_[A from B]_ = 0.4727).

**Table 2:** Mothers’ perception of recovery after treatment of childhood fever by PMDs (N = 385)

	Frequency	Percentage (%)
What happened to the child after treatment by the PMDs		
Fully recovered	143	37.14
Fever persisted	25	6.49
Fever relapsed	102	26.49
Recovered with disability e.g.(inability to walk)	53	13.77
Died	62	16.10
Total	385	100

*PMDs* patent medicine dealers

**Table 3:** Mothers’ view on satisfaction with the PMDs’ treatment of childhood febrile conditions (N = 385)

Satisfaction	Frequency	Percentage (%)	z value	p value
Satisfied	273	70.9	11.604	<0.0002****
Not satisfied	112	29.1		
Total	385	100		

*PMDs* patent medicine dealers

**** p < 0.0001

**Table 4:** Cross tabulation of mothers’ perception of recovery and the satisfaction of mothers after the PMDs treatment of childhood febrile conditions

Question	What is your perception of recovery of your child from childhood febrile condition after treatment by PMDs? Indicate the option below that is most applicable to you					
Mothers’ perception	The child recovered fully	Fever persisted	Fever relapsed	Child recovered with disability	Child died	Total
Satisfied	137 (50.18 %)	18 (6.59 %)	92 (33.70 %)	9 (3.30 %)	17 (6.22 %)	273
Not satisfied	6 (5.36 %)	7 (6.25 %)	10 (8.93 %)	44 (39.29)	45 (40.18 %)	112
Z	8.2680***	0.4560	5.0020**	−9.3090**	−8.2320**	
p	<0.0001	0.6484	<0.0002	<0.0002	<0.0002	
Total	143	25	102	53	62	385

*PMDs* patent medicine dealers

χ^2^ = 192.94, df = 4; p < 0.0001; Cramer’s v = 0.7079, lambda_[A from B]_ = 0.8727, when predicting “mothers’ satisfaction” from a knowledge of “mothers’ perception of recovery, lambda_[A from B]_ = 0.4727, when predicting “mothers’ perception of recovery” based on knowledge of “mothers’ satisfaction”

### Focus group discussions

There were 33 participants for the FGDs, with an age range of 21–47 years. Most of the participants stated the reasons why they were satisfied with PMDs’ treatment of childhood febrile conditions included PMDs’ politeness, caring attitude, drug availability, easy accessibility, flexibility in pricing, shorter waiting time, their God-fearing nature, and disposition as good listeners. They agreed that the attitudes of some health workers were often very impolite, unlike the PMDs. However, some other mothers agreed that some of the health workers were approachable and polite. One mother stated

*“How can I be unsatisfied with the PMDs’ treatment of my child, when I experienced the bad attitudes of the nurses and ward maid (health assistants) at our health centre, for uncountable times? They (nurses and health assistants) talk to us anyhow……, even when I was worried, and cried out my eyes over fears of my child’s survival, because her body has been very hot all night, yet they (nurses and health assistants) did not pity me, rather they shouted severally at me instead of talking to me calmly…….. After a particular bitter experience, I decided never to go back there (PHC) again….. I was not in the mood to be insulted, and so I took my child to the chemist (PMD), who was quite friendly, and behaved as if he was the father of my son. Since then, I stopped taking my child to the health centre.”*

Some mothers felt that the health staff should not be blamed if they get irritated because many staff are available to attend to crying babies and their distraught mothers during health emergencies.

Another mother said that she was *satisfied with PMD’s treatment of her child, because the PMD in her area is kind, has a good heart and treats her family like one of his household.*

Another mother stated that she was satisfied with PMD’s treatment of her child’s febrile conditions because of the *“exceptional care”* her sick child received each time she patronized the PMD. The woman stated—

*“on a certain occasion…I went to the health centre in the dead of the night, and the ward maids (health assistants) told me that there was no one to help my child. I became afraid that she might die, and rushed her to the house of the chemist (PMD) who lives near the market square. On hearing my cries, he woke up, opened his doors to me, and even bathed my daughter who also had running stomach (diarrhoea). My daughter got better after the chemist’s (PMD’s) treatment. The chemist (PMD) provided us a place to sleep till day break so that he could watch over my daughter.”*

Most of the participants had similar stories of their encounter with PMDs and contrasted it with their experiences with local health staff. Other participants agreed that although the local health centre was nearer their homes than the PMD shops, they were preferred visiting the PMDs because of shorter waiting time. Another mother stated that her children had been treated for childhood febrile conditions at various times by PMDs who never turned them away even when she had no money to pay for the services.

On the perceived recovery of their child after treatment by PMDs, some of the mothers perceived that their child’s recovery was sometimes slow or delayed, while some of the participants felt their child had prompt recovery, which they attributed to the fact that the PMDs always gave them many drugs, unlike staff of the PHC where the drugs were fewer, and therefore not as effective. One of them said that

*“even for a small febrile condition, the chemist (PMD) mixes many good drugs for my child, and will mix these drugs according to the amount I can afford.”*

Most of them stated that they were always satisfied with the PMDs because they can tell whether the drugs their child needs are available, unlike at the local health centre. Another mother agreed that although her child was unable to walk for some days, when the child recovered from a severe episode of fever, after treatment by the PMD, she was not worried, because the PMD gave her assurances that once the *“bad blood”* that caused the problem *“melts away”* the child will regain his walking ability.

Most of the mothers who shared experiences of poor treatment outcome after PMDs’ treatment, believe that the problem is not that the PMDs are not knowledgeable in diagnosis and management of childhood febrile conditions, but rather they believe the government has allowed an influx of adulterated drugs into the country and the PMDs may unknowingly give fake drugs which will not *“work”* to bring about a faster recovery. Some other participants disagreed, and expressed the view that PMDs have limited knowledge of drugs and diseases, unlike health workers; they want the government to train PMDs on how to use *“good English (orthodox) medicine”* to treat their children during fever episodes.

Most participants stated that PMDs are God fearing, and some PMDs usually pray for their child before giving them medication, and such prayers make the medicine more effective. In a mother’s view:

*“the chemist (PMD) is God’s answer to the cry of poor rural women, especially poor widows, many of whom would have lost their child to various febrile illnesses, if not for the heroic services of the chemist (PMD). Whenever the chemist (PMD) prays for my child before giving medication, I see the instant effect that God is at work almost immediately, because my child will start sweating”*

As one mother stated:

*“we (the women) will continuously patronize the chemist (PMDs) as long as our children have febrile conditions or even other illnesses, because they understand us and we understand them. They do not treat us with arrogance as if we are animals. They laugh when we laugh and cry with us when we cry. What else can we ask of them?”*

## Discussion

This study revealed that fewer than half of the mothers who resorted to PMDs for treatment of childhood febrile conditions perceived that their child had fully recovered. This result is not unexpected considering there are various aetiological factors for febrile conditions, which may be difficult for PMDs to diagnose and manage successfully. For instance, this study showed that 25 (6.5 %) mothers perceived that their child’s fever had persisted despite treatment by PMDs. This situation can arise when there is misdiagnosis and/or mismanagement of a childhood febrile condition by PMDs. This possibility gives credence to the findings of another study [11] that any illness presenting with fever was taken and treated as malaria by PMDs.

### Strengths and weaknesses of the study

The present study holds strength in the large sample size that was studied. Mothers’ perception of recovery and mothers’ satisfaction after treatment of childhood febrile conditions by PMDs was simultaneously investigated in a very large and well-described rural population, which might be relevant in understanding mothers’ health-seeking behaviour in mostly poor populations. The community-based design allowed simultaneous presentation of views/opinions from mothers with different experiences after PMDs’ treatment of childhood febrile conditions in rural areas, controlling for key factors, including location of home (rural *vs* urban), parity and age of child when treated by PMDs. However, the study is not without limitation, because the cross-sectional nature of the study means that it is difficult to infer causality between PMDs’ treatment of childhood febrile conditions and either mothers’ satisfaction with treatment or mothers’ perception of recovery of their child. Moreover, mothers’ satisfaction with PMDs’ treatment and mothers’ perception of recovery of their child after PMDs’ treatment, were self-reported, which may limit the accuracy of measurements. In addition, it is difficult to infer whether mothers’ satisfaction with PMDs’ treatment in childhood febrile conditions was affected by mothers’ experiences with PMDs’ treatment of other non-febrile conditions, and whether such treatments were preceded by PMDs’ treatment of childhood febrile conditions. Over time, as a child grows to 5 years old, longitudinal observations on mothers’ satisfaction with treatment or mothers’ perception of recovery of their child after PMDs’ treatment would be possible, but were not explored in this study. Mothers’ satisfaction with treatment and mothers’ perception of recovery of their child from childhood febrile conditions, as behavioural responses to PMDs’ treatment, were self-reported and not objectively measured. However, the test instrument validated self-reporting, which allows these measures to be applied to large numbers of people. Perception and satisfaction are established influential factors on consumer behaviour, and these results have translational importance on health-seeking behaviour. In spite of these limitations, the strengths of the study suggest that it has both scientific and practical implications.

### Relevance of findings to the field

The stereotypical, and often wrong, diagnosis on which the treatment of childhood febrile conditions are based by PMDs may probably explain why some mothers perceived that their child’s fever had persisted, relapsed, child recovered with disability or child died, despite PMDs’ intervention. This was corroborated in the FGDs where most mothers agreed that their child did not recover fully, and some others stated that their child recovered with loss of function, after treatment by the PMDs. These findings suggest an unacceptable state of affairs. The reasons for this treatment failure have been identified by mothers in the FGDs as mainly due to unintentional use of fake drugs and limited knowledge of the right drugs and their prescription. This corroborates an earlier study [14], which reported that treatment given by PMDs could be ineffective due to drug overdose or underdose and its attendant consequences, such as anaemia, encephalitis and cardiac failure, among others. These adverse situations may arise since it has been observed [15] that PMDs do not have formal health training, and hence incidences of drug overdose or underdose and attendant consequences are quite high in their treatment practice. It is plausible that mothers’ perception that their child recovered with disability or died after PMDs’ treatment of childhood febrile conditions, might be related to either mismanagement or misdiagnosis by PMDs. Another study [11] that interviewed PMDs, in Ugwogo-Nike, on their treatment practices, observed widespread incorrect diagnosis and drug prescription. The study revealed that PMDs’ treatment practices in Ugwuogo-Nike included the administration of anti-pyretics, analgesics, anti-malarials, and incomplete dose of antibiotics depending on the purchasing power of the customer, and not the appropriate drug to treat the illness. This finding was corroborated in the present study, as in the FGDs some mothers stated that the PMDs gave them drugs according to what they could afford.

Okeke et al. [11] revealed that PMDs were aware of the drugs Coartem, Halfan and mefloquine, but rarely procured/dispensed them because they were considered too expensive for their rural clients, and dispensed instead cheaper alternatives, which may be fake [16]. It is possible that PMDs’ treatment practices at Ugwuogo-Nike are built upon profitable business models and lacked professional, altruistic standards. The danger is that most patients treated with incomplete doses may harbour drug-resistant strains of microbes that could lead to subsequent treatment failure. This practice is unlikely to change since most PMDs have no pharmaceutical training and sell drugs on a retail basis for profit [4, 17], and, perhaps, lack the guidance in relevant ethical requirements.

### Implications for care teams and policymakers

Despite all the disadvantages and that the majority of mothers perceived that their child did not recover fully after patronizing PMDs, yet the majority of mothers were satisfied with PMDs’ treatment in childhood febrile conditions. Although 112 (29.1 %) mothers were not satisfied, a significant proportion was satisfied. These findings agree with earlier studies [1, 18], which showed that a majority or 77.5 % of those who sought care from PMDs were satisfied with the first-line of treatment, and did not seek further treatment. This trend was corroborated by results from the FGDs that revealed, despite negative treatment outcomes, the majority of mothers were satisfied with PMDs’ treatment of childhood febrile conditions. It suggests that this trend may represent a reflection of mothers’ positive perception of PMDs’ services and not necessarily treatment outcome. Similarly, when the proportions of mothers who reported that their child died and those who perceived that their child recovered with disability, are summed up, it was determined that 115 (29.9 %) of mothers perceived a negative treatment outcome. The near-equal proportions of mothers that were not satisfied with PMDs’ intervention in childhood febrile conditions (112, 29.1 %) and those that perceived their child recovered with disability or died (115, 29.9 %) could suggest a link between lack of satisfaction and perception of recovery by mothers. Further, it was revealed that the greatest proportion of mothers who were dissatisfied with PMDs’ treatment, perceived that their children died after treatment by PMDs. The proportion of mothers who perceived that their child recovered fully was significantly higher among those that were satisfied with PMDs’ treatment than those who were not. Contrary to expectations, the data analysis showed that 87.77 % of errors in predicting the relationship between mothers’ perception of recovery and mothers’ satisfaction can be reduced by taking into account mothers’ perception of recovery of their child in response to PMDs’ treatment, whereas only 47.77 % of errors in predicting the relationship between mothers’ satisfaction and mothers’ perception of recovery of their child can be reduced by taking into account mothers’ satisfaction with the PMDs’ treatment in childhood febrile conditions. It provides supportive evidence that there is weak accord between mothers’ satisfaction and mothers’ perception of recovery of their child from febrile conditions after treatment by PMDs. It suggests the possibility that client satisfaction, which may influence uptake of healthcare services, may not be solely determined by perceived treatment outcome, but by intervening variables, such as incentives that are offered by service providers.

The PMDs’ strength in this regard are highlighted in previous studies [1]. Mothers’ satisfaction with PMDs’ treatment in childhood febrile conditions observed in this study is unlikely to be fully explained by their perception of treatment outcome alone. Otherwise the results of this study would not have shown that the majority of mothers were satisfied with PMDs, including some of the mothers whose child either died or recovered with disability. For instance, it does not make sense that a mother would be “satisfied” with the PMDs’ treatment if their child died or recovered with disability.

It might be beneficial to adopt and replicate the PMDs’ business model in PHCs to improve mothers’ satisfaction with PHCs’ service delivery and influence mothers’ health-seeking behaviour and ensure patronage of PHCs in childhood febrile conditions. These findings set an important agenda to explore how PHCs can offer incentives that might influence mothers’ satisfaction with their services in rural areas.

Overall, mothers’ perception and mothers’ satisfaction with PMDs’ treatment of childhood febrile conditions as measured by the test instrument should be interpreted with caution because mothers relied on a long period of memory recall to provide responses. However, the results of the FGDs appear to validate the results obtained with the test instrument. It does appear that the test instrument was reliable, and to a reasonable extent, recorded major trends in mothers’ perception of recovery and mothers’ satisfaction with PMDs in the rural community.

## Conclusions

Mothers’ satisfaction could be the key driver of mothers’ health-seeking behaviour and is less likely to be influenced by mothers’ perception of recovery of their child. Mothers’ negative perception of their child’s recovery may not induce proportionate change in mothers’ health-seeking behaviour, which seems to be influenced mainly by mothers’ satisfaction with the positive attributes of PMDs’ personality/practice, which sets an important agenda for PHC reforms.
